# Goosecoid Promotes the Metastasis of Hepatocellular Carcinoma by Modulating the Epithelial-Mesenchymal Transition

**DOI:** 10.1371/journal.pone.0109695

**Published:** 2014-10-24

**Authors:** Tong-Chun Xue, Ning-Ling Ge, Lan Zhang, Jie-Feng Cui, Rong-Xin Chen, Yang You, Sheng-Long Ye, Zheng-Gang Ren

**Affiliations:** 1 Liver Cancer Institute, Zhongshan Hospital, Fudan University, Shanghai, P.R. China; 2 Key Laboratory of Carcinogenesis and Cancer Invasion (Fudan University), Ministry of Education, Shanghai, P.R. China; Seoul National University, Republic of Korea

## Abstract

The homeobox gene, *goosecoid* (*GSC*), is a transcription factor that participates in cell migration during embryonic development. Because cell migration during development has characteristics similar to cell invasion during metastasis, we evaluated the potential role of GSC in the metastasis of hepatocellular carcinoma (HCC). GSC expression in HCC cell lines and tissues was evaluated, and its effects on the migration potential of HCC cells were determined by GSC knock-down and overexpression methods. In addition, the prognostic role of GSC expression in the metastasis of cancer cells in HCC patients was determined. Our data showed that GSC was highly expressed in several HCC cell lines, particularly in a highly metastatic HCC cell line. Overexpression of GSC promoted cell migration and invasion of HCC cells *in vitro*. Gain-of-function induced the epithelial-mesenchymal transition but not collective cell migration, whereas loss-of-function induced the reverse change. High-level expression of GSC correlated closely with poor survival and lung metastasis in HCC patients; lung metastases showed more upregulated GSC expression than the primary tumor. We conclude that GSC promotes metastasis of HCC potentially through initiating the epithelial-mesenchymal transition. GSC is also a prognostic factor for poor survival and metastasis of HCC, which suggests its potential as a therapeutic target for metastatic HCC.

## Introduction

To date, metastasis is still one of the main obstacles to the survival of patients with hepatocellular carcinoma (HCC) [1], . Tumor cells have evolved traits that allow them to disseminate and travel systemically, and these characteristics are similar to the migration ability of embryonic cells during development.

Cell migration is an important process during embryonic development [3]. There are two types of migration in embryonic development, which include epithelial-mesenchymal transition (EMT) [4], [5] and collective cell migration [6], [7]. EMT is characterized as the shift of cell phenotype from epithelial cells to mesenchymal cells, which have increased ability to migrate. The process of EMT is essential for germ layer formation and cell migration in the early vertebrate embryo, particularly during gastrulation [8]. On the other hand, collective cell migration is characterized as movement of groups of cells by membrane ruffling at the free edge only, while cell-cell junctions within the moving cell group remain intact. This process occurs in the developmental context during gastrulation [9] and formation of the neural crest [10]. Similarly, it has been well demonstrated that EMT takes place during the metastasis of tumors. Meanwhile, collective cell migration also has been shown to play critical roles in invasion and spreading of tumors, such as melanoma. Therefore, the essential genes that control EMT or collective cell migration during embryogenesis may potentially play critical roles in invasion and metastasis of malignancies.

*Goosecoid* (*GSC*), a paired-like homeobox gene expressed in the vertebrate organizer, plays critical roles in both gastrulation [11]–[13] and neural crest development [14]. Therefore, GSC expression is correlated with both the EMT process and collective migration. Accumulated evidence has shown that GSC induces morphogenetic movements during gastrulation and controls cell migration in Xenopus embryos [15], which suggests that the target genes controlled by the *GSC* DNA-binding protein might include genes involved in intercellular signaling, cell motility, and cell adhesion. In Xenopus embryos, GSC-controlled cell movement affects migration of groups of cells but not of individual cells. However, another report suggested a role for GSC in promoting metastasis of human breast tumors through initiation of EMT [16]. Therefore, the potential role of GSC in other malignancies remains to be uncovered. It has been shown that EMT is correlated closely to the invasion and metastasis of HCC; however, the underlying molecular mechanisms controlling the behavior of HCC tumor cells remain unclear. The finding that GSC controls the metastasis of breast tumors, which are derived from an epithelial source, has led us to investigate in the potential roles of GSC in migration of HCC cells.

Herein, we evaluated the expression of GSC in HCC cell lines with different metastatic potential. Wound-healing and Matrigel invasion assays were used to evaluate the function of GSC in HCC cell movements. Gain-of-function and knock-down of *GSC* in HCC were used to further explore the potential mechanism of GSC in this process. The prognostic role of GSC in extra-hepatic metastasis and survival of human HCC after hepatic resection also was evaluated.

## Materials and Methods

### Cells lines

Human HCC cell lines with elevated lung metastasis potential (namely, MHCC97L, MHCC97H, and HCCLM3) were established at the Liver Cancer Institute of Fudan University. The human HCC cell lines with low metastatic potential that we evaluated were SMMC-7721 (established at Second Military Medical University), Hep3B, and HepG2 (obtained from American Type Culture Collection). L02, an immortalized human liver cell line, was obtained from the Chinese Scientific Academy. These cell lines were cultured in high glucose DMEM (GibcoBRL, Grand Island, NY) supplemented with 10% fetal bovine serum (Hyclone, Logan, UT).

### Patients and follow-up

A tissue microarray (TMA) composed of samples from 112 HCC patients was used in this study. These patients were retrieved from a prospectively designed database. Paraffin tissue sections were stained by hematoxylin and eosin, and reviewed by two pathologists according to the WHO histomorphologic criteria. Ninety-four patients were positive for the hepatitis B surface antigen (HBsAg). All patients were classified as Child-Pugh A. The follow-up procedures were carried out as described in our previous study [17]. Ethics approval was obtained from the Zhongshan Hospital research ethics committee, and written informed consent was obtained from each patient.

### Quantitative Real Time RT-PCR

Real-time reverse transcription-PCR (RT-PCR) was established using Taqman PCR reagents and ABI PRISM 7700 sequence detection system (Applied Biosystems, Foster, CA) in accordance with the protocol described previously [18]. The primers used for GSC amplification were described previously [16]. The assay was performed in triplicate, and the results were analyzed using Student's *t* test.

### Western blot

Western blotting was performed according to the protocol of Bio-Rad wet transfer using the Bio-Rad Transfer Cell System (Bio-Rad, Ontario, Canada). Mouse anti-human GSC IgG (Abcam, Cambridge, MA) 1∶500, rabbit anti-human E-cadherin mAb 1∶1000, N-cadherin 1∶1000, β-catenin 1∶2000, vimentin 1∶800 (Cell Signaling Technologies, Danvers, MA), and rabbit anti-human β-actin mAb (Epitomics, Burlingame, CA) 1∶1000 were used as primary antibodies in detection. Horseradish peroxidase-conjugated goat anti-rabbit IgG F(ab′)2 antibody (Jackson ImmunoResearch, West Grove, PA) at 1∶5000 was used as secondary. Photos were analyzed using Image Lab software (Bio-Rad, Ontario, Canada). The relative protein expression levels were normalized to β-actin before comparison.

### Lentivirus constructs and cell infection

Full-length human *GSC* cDNA was subcloned into the LV5-EF1a-GFP/Puro lentivirus vector (GenePharma Corp., Shanghai, China). Viral particles were produced by co-transfection of the shRNA plasmid and the lentiviral packaging plasmid into 293T cells. A corresponding vector containing the GFP gene was used as control. HCC cells were infected with the lentiviral particles, and were selected with 3 mg/mL puromycin (P8833; Sigma-Aldrich). Stably transfected clones were characterized for expression levels of GSC protein using inverted fluorescence microscopy, real time RT-qPCR, and immunoblotting.

### RNA interference

Small interfering RNAs (siRNAs) were synthesized to target expression of GSC (GenePharma Corp., Shanghai, China). The coding sequences were as follows: siGSC-158, 5′-GCAUGUUCAGCAUCGACAATT-3′ (position 158 of GSC mRNA); negative control siRNA, 5′-UUC UCC GAA CGU GUC ACG UTT-3′. Tumor cells were seeded at a density of 5×10^4^ cells in wells of 24-well plates and cultured overnight. SiRNA (20 pmol) was transfected by use of Lipofectamine2000 according to the manufacturer's protocol (Life Technologies, Grand Island, NY). Cells were harvested at 24 h or 48 h post-transfection.

### Measurement of cell proliferation

We measured the proliferation of HepB3-GSC versus HepB3-NC using the Cell Counting Kit-8 (CCK8; Dojindo Molecular Technologies Inc., Kumamoto, Japan) according to the manufacturer's instructions. Cells were counted and stained with Trypan blue staining to determine cell viability; cultures with at least 99% viable cells were used for the CCK8 assay. Briefly, the HCC cell lines were cultured in 96-well culture plates at 3×10^3^cells/well in growth medium for 24, 48, and 72 hours, and then harvested for the CCK8 assay. Results were expressed as the absorbance of each well at 450 nm (OD_450_) as measured using a microplate spectrophotometer (Multiskan Spectrum, Thermo Fisher Scientific, Waltham, MA, USA). The assay was performed in triplicate.

### Wound healing assay

For the scratch assay, cells were grown to confluence in a 6-well plate, and a “wounding” line was scratched into the cell monolayer with a sterile 200-µl pipette tip. The remaining cells were washed twice with culture medium to remove cell debris, and the cultures were incubated at 37°C with 1% serum–containing DMEM culture medium. The width of the wound was measured under a microscope at 0, 24 hours, and 48 hours after the scratch to assess the migration ability of the cells. Wound healing was determined at each time point by calculating the distance migrated relative to the original distance at 0 hour. The assay was performed in triplicate, and results were analyzed using Student's *t* test.

### Matrigel invasion assay

Tumor cell invasion assay was performed as previously [18]. Briefly, using 24-well Transwell chambers, the upper chambers with polycarbonate filters (8-µm pore size; Costar, Acton, MA, USA) were coated with 50 µl of Matrigel (BD Biosciences, San Diego, CA, USA). Cells (1.0×10^3^ in 100 µl DMEM) were collected and added to the pre-coated wells. The cells were allowed to invade toward the lower chamber. The cells migrating to the membrane were enumerated with Giemsa staining. The assay was performed three times. Results were analyzed using Student's *t* test.

### Fluorescence analysis of collective cell migration

Cell-sheet migration assay was performed based on modification of a previous method [19]. Hep3B-GSC cells and Hep3B-NC cells expressing GFP were cultured in six-well culture plates at 5×10^5^cells/well in growth medium and grown to confluence. The cell monolayer was wounded using a sterile pipet tip (∼700 µm in width), and the cells were incubated with 2% serum-containing culture medium. Lines lightly etched with a razor blade on the bottom of the dish provided a reference under the microscope. Dynamic activity of marginal protrusions was visualized every 6 hours by fluorescence microscopy.

### Tissue Micro Array (TMA) and immunohistochemistry

The construction of the TMA and the protocol for immunohistochemistry were described previously [20]. Because the lung is the most frequent target organ of HCC metastasis, the TMA was constructed based on whether or not the patients experienced lung metastasis during the long follow-up period post-resection. Tissues from 112 HCC patients were used to generate the TMA, and all tissues were from the primary HCC lesion. Six lung tissue samples from patients who underwent partial pulmonary resection also were included in the TMA. A two-step immunohistochemistry method that included a heat-induced antigen-retrieval procedure was performed as previously described [21]. Mouse anti-human GSC mAb (Abcam, Cambridge, MA, USA) at 1∶200 was used as the primary antibody. Normal colon tissue was used as the positive control. Normal liver tissue, a hepatic hemangioma sample, and omission of primary antibody were used as negative controls. Two pathologists read the slides independently, and they were blinded to the study design and the patient data. The immunoassay results were defined by the staining intensity and the percentage of positive tumor cells as described previously [22]. Categories of staining (GSC^High^, GSC^Medium^, and GSC^Low^) were established previously [20].

### Statistical analysis

When two groups of cells or animals were compared, analysis was performed using Student's t test. For comparison of multiple groups, a one-way ANOVA was used with Turkey's HSD post-hoc analysis to determine individual group differences. Testing for the homogeneity of variance was performed before the post-hoc analysis. The Pearson Chi-Square test was used to compare qualitative variables in clinical pathology analysis. When expected sample values were below 5, Fisher's exact test was used. Spearman's rank test was used to detect the correlation between variables. Overall survival (OS), relapse-free survival and extra-hepatic metastasis-free survival were determined using the Kaplan-Meier method to describe the survival curves, and the log-rank test was used to compare survival distributions between groups. Univariate and multivariate analyses were based on the Cox Proportional Hazards Regression model. Receiver Operating Characteristic curve (ROC) was used to confirm the predictive accuracy of risk factors. All P values were 2-tailed, and the statistical significance was set at *P*<0.05. Statistical analyses were performed using SPSS18.0 software (SPSS Inc., Chicago, IL).

## Results

### Expression of GSC in HCC cell lines

**Figure 1:**
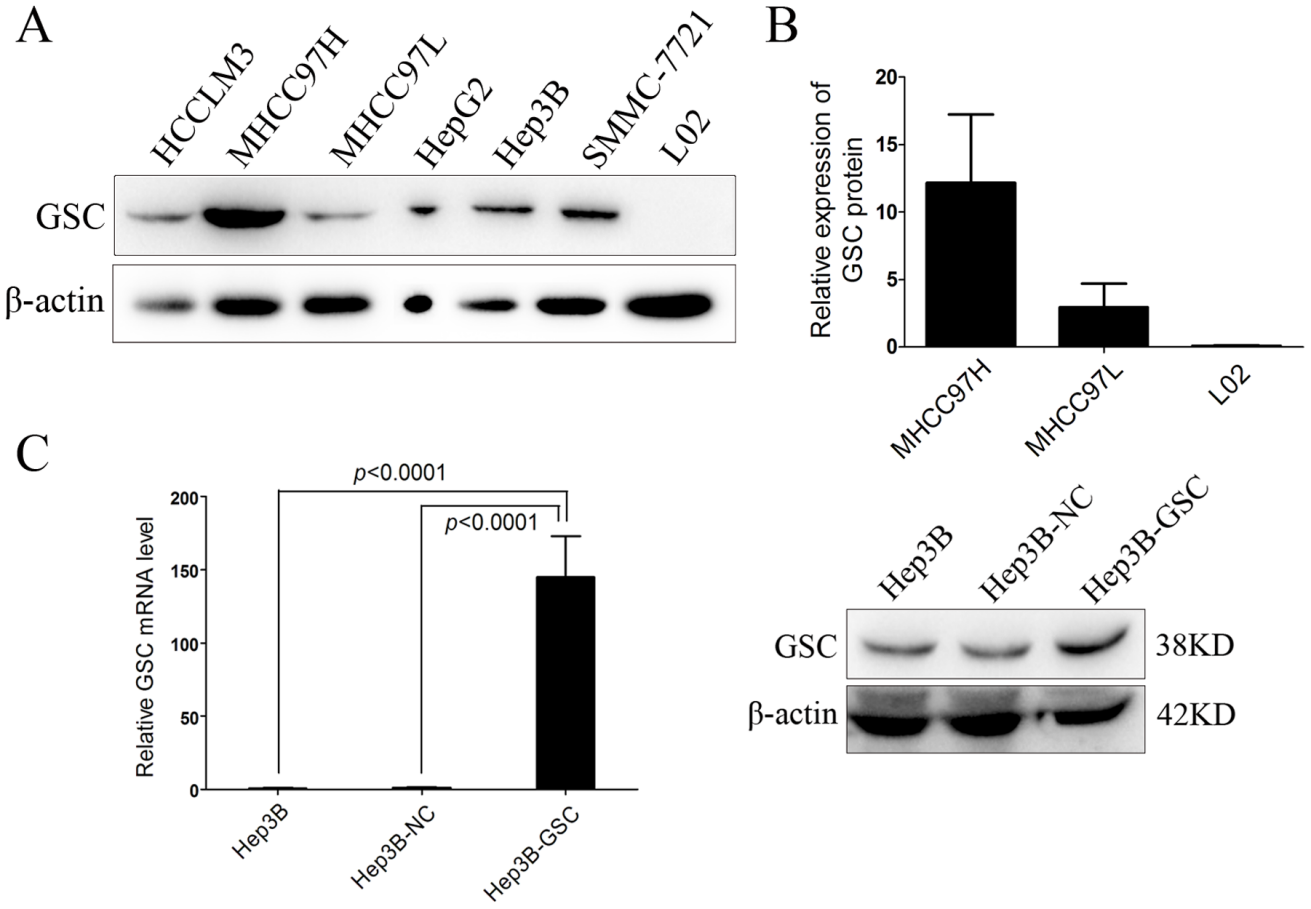
GSC expression in HCC cell lines. (A) Western blot indicates that GSC is abnormally expressed in HCC cell lines, whereas it is not detectable in a normal hepatic cell line. (B) Western blot shows that GSC is strongly expressed in the highly metastatic HCC cell line versus the cell line with low metastatic potential. (C) *GSC* was overexpressed in HCC cells through lentivirus infection. A stable cell line was selected with 3 mg/mL puromycin. Quantitative real-time RT-PCR shows the prominent overexpression of *GSC* in Hep3B cells, which was confirmed by western blot. The data are presented as the mean ± SD of at least three independent experiments.

To measure GSC expression in HCC, several HCC cell lines were selected including HCCLM3, MHCC-97H, MHCC-97L, HepG2, Hep3B, SMCC-7721 and an immortalized liver cell line. The HCC cell lines showed upregulated GSC expression compared to the L02 cell line (Figure 1A). In addition, highly metastatic MHCC97H cells had stronger GSC expression than the low metastatic potential MHCC97L cells, which have a genetic background that is similar to MHCC97H (Figure 1B). The Hep3B cell line was chosen for further *in vitro* analyses.

### Highly expressed GSC promotes HCC migration and invasiveness

**Figure 2:**
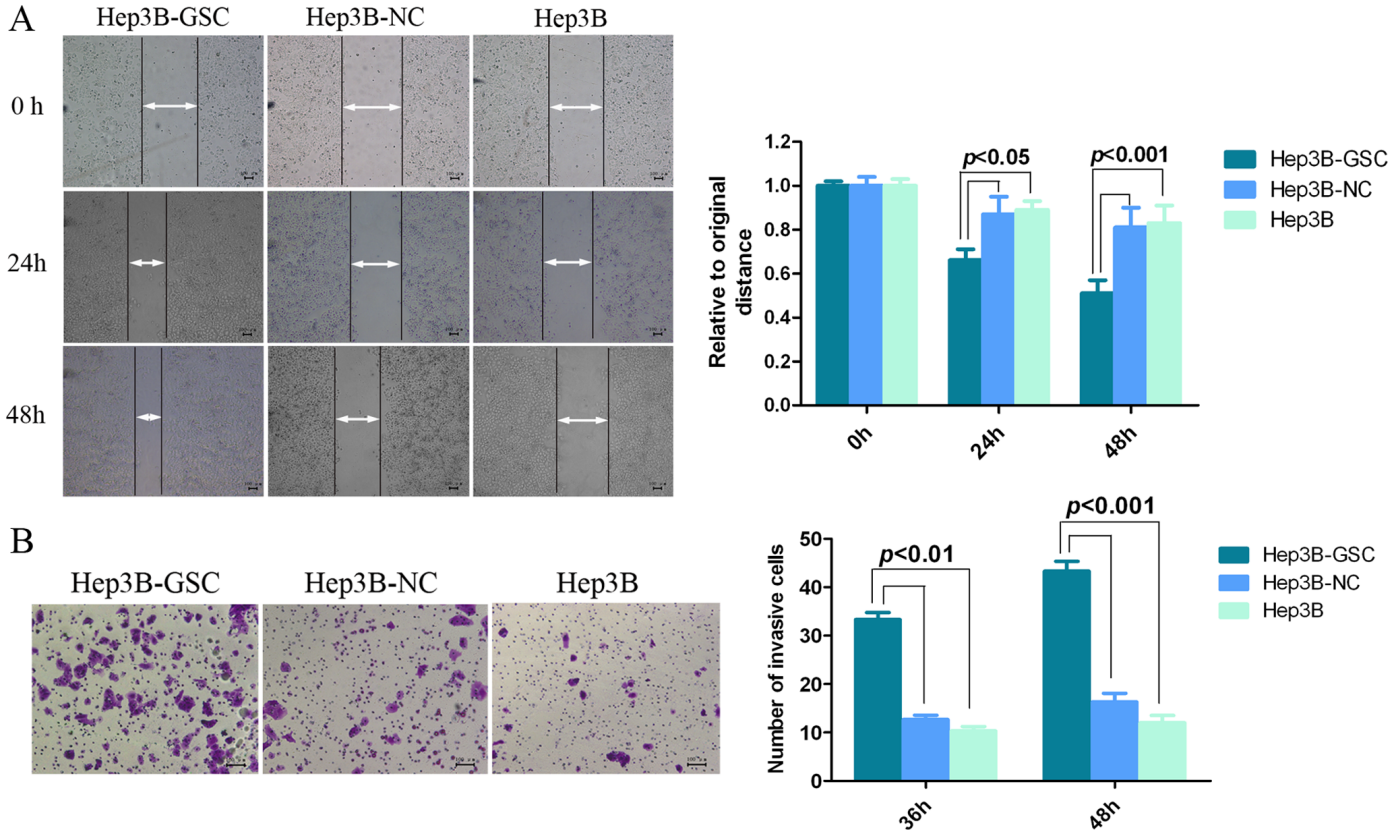
Effects of GSC overexpression on migration of HCC. Hep3B-GSC cells were infected by *GSC* lentivirus and were selected with puromycin. (A) Wound-healing assay shows that Hep3B-GSC cells migrated faster than normal Hep3B or Hep3B cells without treatment after 24 h and 48 h. The cleared area of Hep3B-GSC was less than the controls. (50× original magnification, size bar: 100 µm) Results were analyzed with the Student *t* test. (B) Giemsa staining indicated the number of Hep3B-GSC cells that migrated through Matrigel was higher than Hep3B cells without treatment or treated with negative control (100× original magnification, size bar: 100 µm). The data are presented as the mean ± SD of at least three independent experiments. Results were analyzed using Student's *t* test.

To characterize the role of GSC in HCC, GSC was overexpressed in Hep3B cells by lentivirus infection. Real-time RT-PCR showed the upregulation of *GSC* in Hep3B cells, which was confirmed by the Western blot results (Figure 1C). Results from the wound healing assay indicated that Hep3B cells overexpressing GSC (Hep3B-GSC) migrated faster than the Hep3B-NC (negative control group) at 24 h and 48 h (*P*<0.05) (Figure 2A). Meanwhile, the CCK-8 assay indicated that there was no difference in cell number between the three groups at 48 h (*P*>0.05) (Figure S1). Invasion assay results indicated that the number of Hep3B-GSC cells that migrated through the Matrigel were significantly greater than the number of Hep3B-NC and Hep3B cells at 36 h (*P*<0.01) and 48 h (*P*<0.001) (Figure 2B).

### GSC increases the migration potential of HCC cells, which is mediated through EMT

Early research in development indicated that GSC controls cell migration in *Xenopus* embryos, and that GSC-controlled cell movement affects migration of groups of cells [15]. On the other hand, recent research suggested that GSC increased the metastatic potential of breast cancer cells by initiating EMT [16]. The gain-of-function assays suggested the potential role of GSC in HCC metastasis; therefore, we explored whether this phenomenon also occurs in HCC.

**Figure 3:**
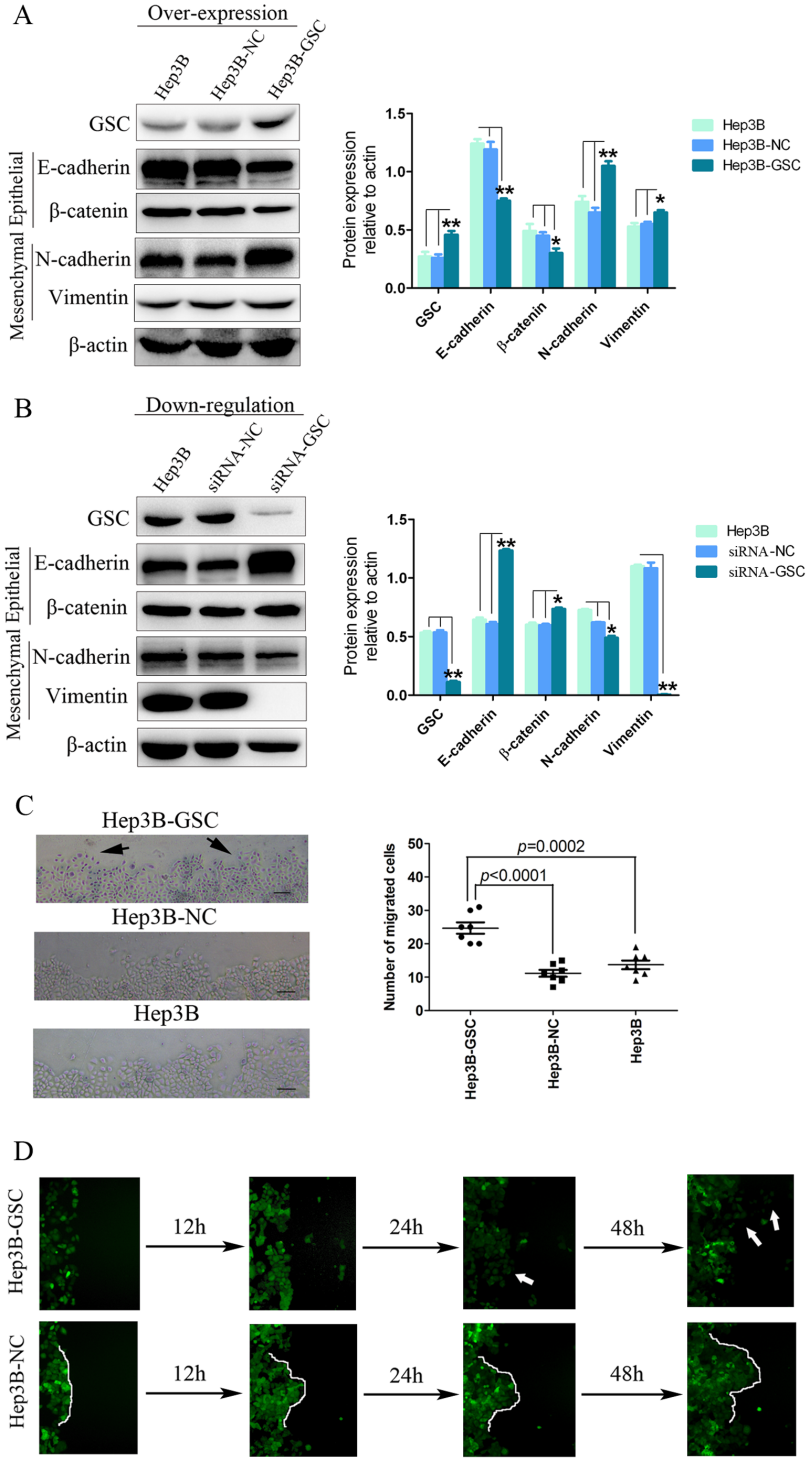
GSC induces EMT but not collective cell migration of HCC cells. (A) Ectopic expression of GSC in Hep3B cells induced changes in expression of EMT markers, particularly E-cadherin. The expression of each protein was normalized to β-actin. Results were analyzed using Student's *t* test. (*, *P*<0.05; **, *P*<0.01) ANOVA analysis also indicated significant differences among the three groups (*P* = 0.001 for GSC, *P*<0.001 for E-cadherin, *P* = 0.004 for β-catenin, *P*<0.001 for N-cadherin, and *P* = 0.007 for vimentin). In addition, post hoc analysis confirmed the significant difference between Hep3B-GSC cells and Hep3B cells without treatment or treated with negative control. (B) Downregulation of GSC in Hep3B cells reversed the changes in expression of EMT markers as detected by Western blot. Results were analyzed using Student's *t* test. (*, *P*<0.05; **, *P*<0.01) ANOVA analysis also indicated significant differences among the three groups (*P*<0.001 for GSC, *P*<0.001 for E-cadherin, *P*<0.001 for β-catenin, *P*<0.001 for N-cadherin, and *P*<0.001 for vimentin). Furthermore, post-hoc analysis confirmed the significant difference between Hep3B-GSC cells and Hep3B cells without treatment or treated with negative control. (C) The scattering cells (black arrow) in the Hep3B-GSC group were prominently increased compared to the control groups. Continuous counting of seven fields of individual migrating cells showed higher numbers of Hep3B-GSC cells migrating than the Hep3B and the Hep3B-NC groups. (D) Fluorescence micrographs of Hep3B cells expressing GSC or GFP control. Hep3B-GSC cells showed decreased cell-cell contact and strong migration of the individual type (white arrows) under continuous observation. Hep3B-NC cells showed characteristic integrated migration (white contour line). (100× original magnification, size bar: 100 µm).

In the Hep3B-GSC cell line, expression of E-cadherin was decreased (compared with Hep3B *P*<0.001 and Hep3B-NC *P*<0.001, respectively) and β-catenin was decreased (compared with Hep3B *P* = 0.004 and Hep3B-NC *P* = 0.013, respectively); whereas N-cadherin was increased (compared with Hep3B *P*<0.001 and Hep3B-NC *P*<0.001, respectively) and vimentin expression was increased (compared with Hep3B *P* = 0.009 and Hep3B-NC *P* = 0.014, respectively), which is suggestive of EMT (Figure 3A). On the contrary, after knocking-down GSC expression in Hep3B cells, E-cadherin expression increased (compared with Hep3B *P*<0.001 and Hep3B-NC *P*<0.001, respectively); whereas vimentin was strongly decreased (compared with Hep3B *P*<0.001 and Hep3B-NC *P*<0.001, respectively), suggesting that the reverse process, MET, was taking place (Figure 3B). Similar results were observed in the MHCC97H cell line when GSC expression was downregulated (data not shown). Hep3B-GSC cells, which showed decreased E-cadherin expression, also exhibited decreased cell-cell contact during migration and increased disseminating potential. Cell counts confirmed the increased numbers of single or isolated Hep3B-GSC cells versus controls (*P*<0.001) (Figure 3C).

We also evaluated the potential role of GSC in the collective migration of HCC cells. Hep3B-NC cells showed evidence of collective migration as detected by fluorescence microscopy. Furthermore, Hep3B-GSC cells did not have an increase in collective migration ability. In contrast, overexpression of GSC in Hep3B cells induced the migration of individual cells versus groups of cells (Figure 3D).

### GSC expression and clinico-pathologic features

To confirm the high expression of GSC in HCC cell lines, immunohistochemistry analysis was performed using tumors and tissue adjacent to tumor. Of 112 tumor tissues, GSC was detected in most cases (105/112). Meanwhile, 55% (62/112) HCC tissues expressed relatively strong GSC staining. However, in only one case was GSC detected in tissue adjacent to tumor. The intra-tumor expression of GSC was significantly stronger than expression in tissue surrounding the tumors.

**Figure 4:**
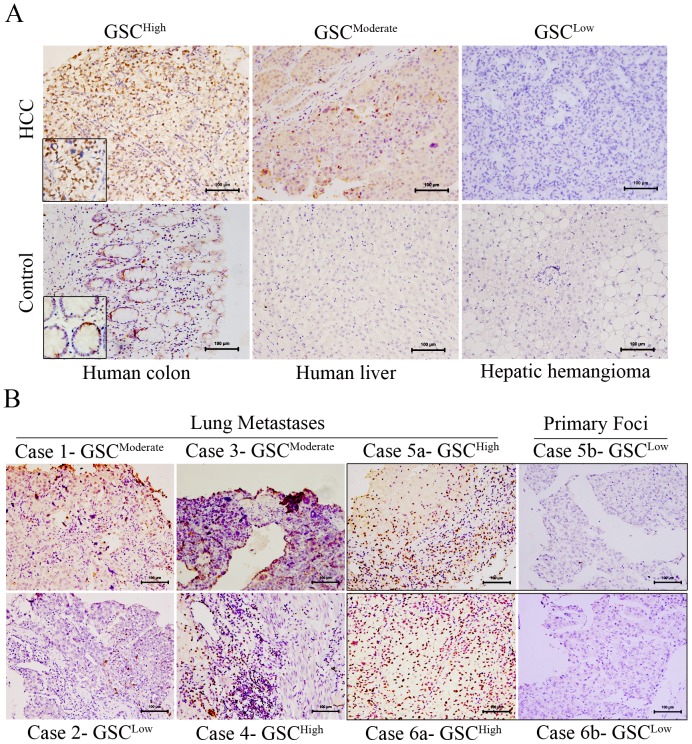
GSC expression in HCC tumor tissues. Immunohistochemistry analysis was performed based on tissue microarray. (A) Top-row shows the staining of GSC from strong to low. Bottom-row shows the controls to confirm the specificity of the GSC antibody. Colon tissue staining was used as the positive control. (B) Six tumor tissues in lung metastases from HCC showed the highly expressed GSC. Comparison of two paired tissues (case 5 and case 6) from lung metastatic cancer and primary foci showed the upregulated GSC expression in lung tissues. (200× original magnification, size bar: 100 µm).

In the positive control, GSC staining was localized mainly to the nucleus of HCC cells (Figure 4A). The positive nuclear expression in colon tissue, negative expression in normal liver tissue or in hepatic hemangioma indicated the specificity of the anti-GSC monoclonal antibody. Patients were stratified according to GSC staining intensity of tumor samples into three groups. GSC^High^, GSC^Medium^, and GSC^Low^ staining was observed in 24, 38, and 50 patients, respectively. There was no significant difference in clinico-pathologic data between the three groups of patients except with respect to lung metastasis (*P* = 0.009) (see Table S1), indicating the strong relationship between GSC expression and distant metastasis in HCC.

### Higher expression of GSC in lung metastatic foci of HCC

Six tumor samples from lung metastatic foci of HCC were retrieved from the followed-up database. These tumor tissues were confirmed histologically to have metastasized from HCC. As shown in Figure 4B, five tissues were GSC positive, including three tissues with highly expressed GSC. Furthermore, the comparison between two paired lung metastatic foci to primary liver cancer indicated significant upregulation of GSC expression in lung metastatic foci.

### Highly expressed GSC is associated with poor survival and lung metastasis in HCC

**Figure 5:**
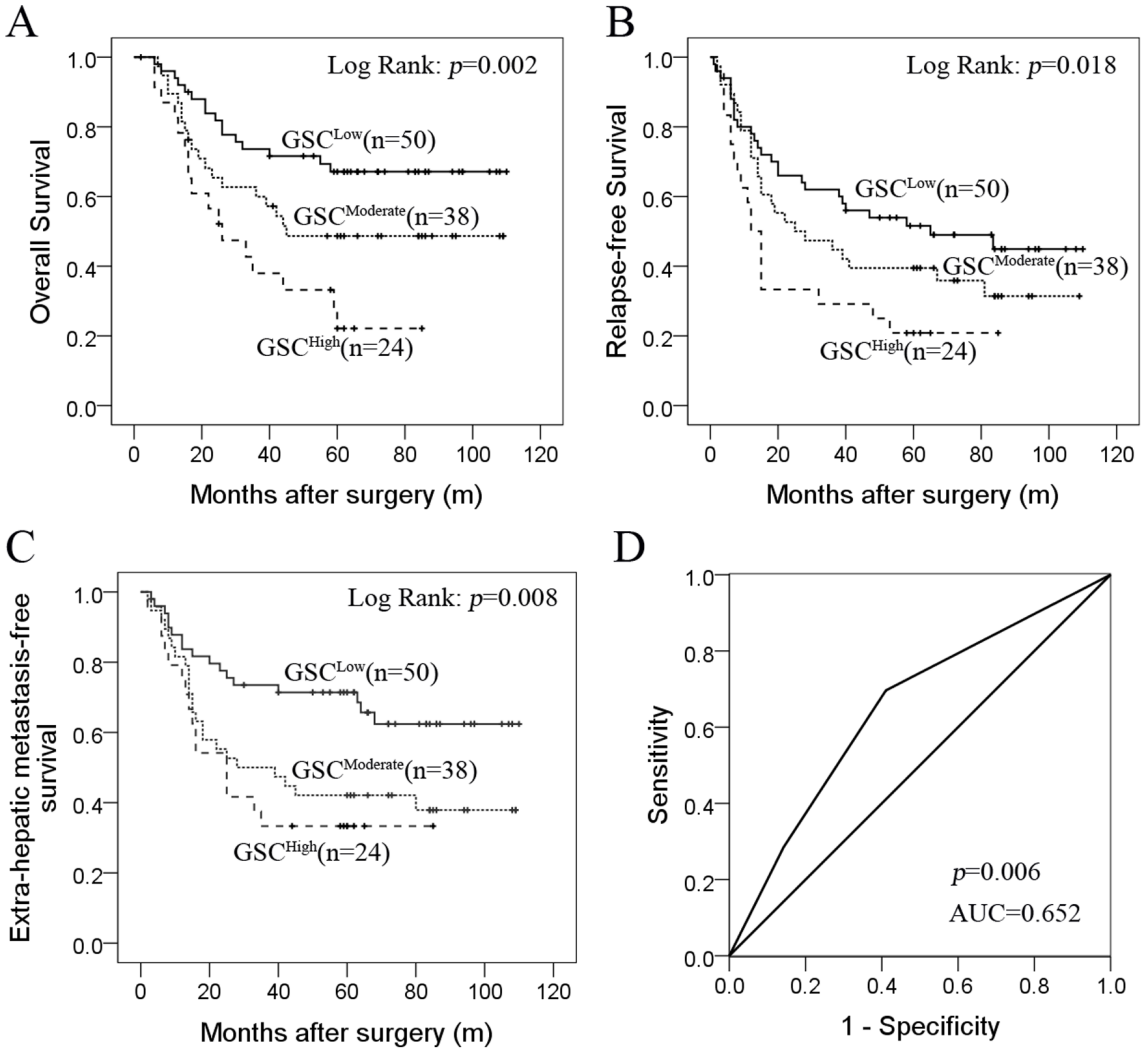
GSC expression correlates with poor survival and lung metastasis in HCC. Immunohistochemistry assay was based on tissue microarray from 112 tumor tissues. (A) Patients with highly expressed GSC had a significantly worse 5-year survival (Kaplan-Meier, log-rank test). (B) Patients with highly expressed GSC had showed early relapse and poor relapse-free survival when compared to other subgroups (Kaplan-Meier, log-rank test). (C) Groups with highly expressed GSC had lower extra-hepatic metastasis-free survival than the group with lower expressed GSC (Kaplan-Meier, log-rank test). (D) Receiver Operating Characteristic (ROC) curve analysis of GSC expression for occurrence of lung metastasis showed areas under the curve (AUC) of >0.5 (*P*<0.05).

There was a statistically significant difference in OS (*P* = 0.002, log-rank test) and relapse-free survival (*P* = 0.018, log-rank test) among the three groups (Figure 5A and 5B). The median OS of group GSC^High^ was significantly shorter than that of group GSC^Medium^ and group GSC^Low^ (26 months versus 45 months and 82 months, respectively).

In group GSC^High^, the cumulative 1-year, 3-year, and 5-year lung metastasis-free survival were only 50%, 20%, and 20%, respectively, whereas in group GSC^Low^, the cumulative 1-year, 3-year, and 5-year rates were 76%, 49%, and 45%, respectively. Time to development of lung metastasis for group GSC^High^ was shorter than for group GSC^Low^ (*P* = 0.008, log-rank test) (Figure 5C). The median time for developing lung metastasis for group GSC^High^ was significantly shorter than for group GSC^Low^ (25 months versus 78 months).

### GSC is an independent prognostic factor for time-to-lung metastasis of HCC

High AFP level, multiple tumor nodules, microvascular invasion, poor tumor differentiation, portal lymphatic invasion, and high GSC expression were all found to be associated with shorter time to lung metastasis by univariate analysis (Table 1). Cox proportional hazards analysis indicated that highly expressed GSC was an independent prognostic factor for shorter time-to-lung metastasis (hazard ratio = 1.842, *P* = 0.001) (Table 1). Group GSC^High^ had nearly twice the likelihood of metastasis to lung than group GSC^Low^. ROC analysis further confirmed the value of GSC as a potential prognostic factor for the occurrence of lung metastasis, with AUC 0.652 (95% CI, 0.550–0.754, *P* = 0.006) (Figure 5D).

**Table 1:** Univariate and multivariate analysis of factors associated with survival and lung metastasis in HCC patients.

	Overall survival				Time-to-lung metastasis			
Features		Multivariate				Multivariate		
	Univariate *P*	Hazard		*P*	Univariate *P*	Hazard		*P*
	values	Ratio	95%CI	Value	values	Ratio	95%CI	Value
Age (≤60 vs.>60 years)	0.887			NA	0.917			NA
Gender (male vs. female)	0.403			NA	0.328			NA
HBsAg (positive vs. negative)	0.726			NA	0.273			NA
Cirrhosis (present vs. absent)	0.721			NA	0.539			NA
AFP (≤20 vs.>20 µg/L)	**0.008**	2.085	1.065–4.084	**0.032**	**0.011**			**NS**
Tumor size (≤5 vs.>5 cm)	0.237			NA	0.278			NA
No. tumor nodules	0.200			NA	**0.096**	2.066	1.068–3.994	**0.031**
(single vs. multiple)								
Tumor capsule	0.348			NA	0.136			NA
(complete vs. none)								
Microvascular invasion	**<0.001**	2.690	1.522–4.755	**0.001**	**<0.001**	2.343	1.306–4.202	**0.004**
(positive vs. negative)								
Tumor differentiation	0.103			NA	**0.058**	2.363	1.338–4.171	**0.003**
Portal lymphatic status	**0.004**	3.837	1.552–9.486	**0.004**	**0.003**	3.064	1.328–7.073	**0.009**
(yes vs. no)								
GSC level	**0.002**	1.685	1.187–2.394	**0.004**	**0.008**	1.842	1.286–2.639	**0.001**
(high vs. medium vs. low)								

Abbreviations: AFP, alpha fetoprotein; ALT, alanine aminotransferase; NA, not adopted; NS, not significant.

In addition, univariate analysis showed that high AFP level, microvascular invasion, portal lymphatic invasion, and high GSC expression were associated with worse OS (Table 1). Moreover, Cox analysis indicated that high GSC expression was an independent prognostic factor for worse OS (hazard ratio = 1.685, *P* = 0.004) (Table 1).

## Discussion

This study is the first to demonstrate that expression of the embryonic gene *GSC* is associated with the metastasis of HCC. First, we determined that GSC was strongly expressed in HCC cell lines, particularly in the highly metastatic HCC cell line. GSC expression in tumors was prominently higher than in peri-tumoral tissue. Overexpression of GSC promoted the migration and invasion ability of HCC cells. Furthermore, GSC expression was correlated closely with extra-hepatic metastasis and poor survival of HCC patients. Importantly, high expression of GSC was shown to be an independent prognostic factor of lung metastasis. These findings suggest the critical role of GSC in metastasis of HCC. Similar to our findings, GSC has been demonstrated to promote metastasis of breast tumors [16]. Therefore, this study further supports the value of GSC as an overexpressed embryonic gene that has a critical role in tumor metastasis in HCC.

In this study, overexpression GSC in HCC cells induced the EMT whereas downregulation GSC inhibited the EMT, suggesting that EMT is one of the potential mechanisms through which GSC promotes metastasis of HCC. Our observation that GSC participates in HCC metastasis through inducing EMT is in concordance with a similar report in breast cancer [16]. On the other hand, HCC-GSC cells showed scattering-type motility whereas control HCC cells exhibited more collective migration, which suggests that highly expressed GSC modifies the migration character of HCC cells from collective cell migration transition to EMT. During embryonic development when neural crest cells separate from their surrounding tissues, their delamination involves partial or complete EMT, then collective migration, which is affected by many positive and negative regulators [23], [24]. It has been shown that GSC expression correlates closely with the function of neural crest cells [14]. Thus, the role of GSC and its relationship to type of migration of HCC cells may be modified by positive and negative regulators in the context of HCC. Our future direction is to evaluate the potential regulators that modify GSC function. Hepatocyte growth factor, which plays a critical role in the metastasis of HCC [25], [26] has been reported to induce not only scattering but also collective migration of colorectal adenocarcinoma cells [27]. In addition, the TGF-β superfamily, including TGF-β or the Nodal family of proteins, has been shown to induce the upregulation of GSC in invasive breast cancer cells and during embryonic development [16], [28]. Thus, these regulators play potential roles in GSC function in HCC.

Published research has indicated that the upregulation of GSC occurs quite early in multistep cancer progression rather than concurrently with the overt display of the invasive phenotype. For instance, it has been postulated that, in ductal-type breast tumors, GSC primes cells for the expression of aggressive phenotypes [16]. In this study, however, it was the lung metastases that showed the highest expression of GSC. In addition, the paired IHC results confirmed the upregulated expression of GSC in metastatic tissues compared to the primary foci. Therefore, it is possible that GSC was upregulated during the late metastatic stage in HCC. In addition, GSC is a member of the homeobox gene family and accumulated evidence suggests that deregulated homeobox gene expression may promote oncogenesis. Meanwhile, the abnormal expression of homeobox genes may be the consequence of wrong cellular context in cancer [29]. Therefore, the upregulation of GSC in metastatic lung tissues may the consequence of a deregulated local microenvironment, which deserves further research.

Taken together, findings from the present study indicate that the embryonic gene GSC is abnormally expressed in HCC and its expression is correlated with metastasis. Moreover, GSC potentially promotes metastasis of HCC through EMT rather than via collective cell migration. However, more in-depth studies exploring mechanisms of *GSC*-related cell-cell contact, organization of the actin cytoskeleton, and cell polarization are needed. Our results suggest GSC is a potential therapeutic target for metastatic HCC.

## Supporting Information

Figure S1 **Effects of GSC overexpression on proliferation of HCC cells.** CCK8 assay was used to evaluate the proliferation of tumor cells at 24 h, 48 h, and 72 h time points. No significant difference was observed among Hep3B-GSC, Hep3B-NC, and Hep3B cells. The data are presented as the mean ± SD of at least three independent experiments. Results were analyzed using Student's *t* test.
(TIF)

Table S1 **Clinicopathologic factors and GSC expression in HCC.**
(DOC)
