# Supplementary figures and images for: Goosecoid Promotes the Metastasis of Hepatocellular Carcinoma by Modulating the Epithelial-Mesenchymal Transition
Source: PLoS One. 2014 Oct 24;9(10):e109695. doi: 10.1371/journal.pone.0109695 (PMC4208742; doi:10.1371/journal.pone.0109695)

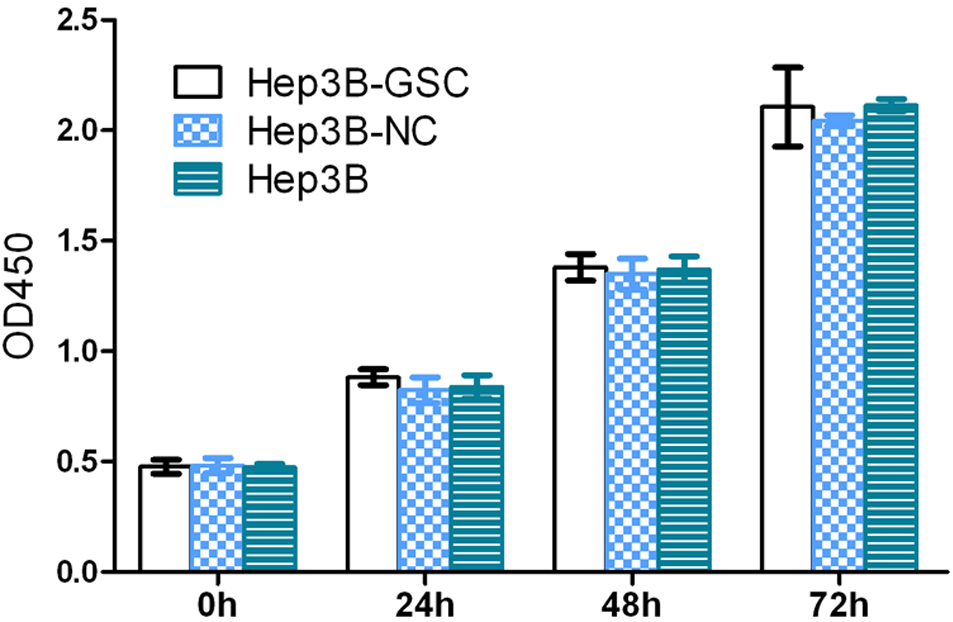

Supplement: Figure S1 — Effects of GSC overexpression on proliferation of HCC cells. CCK8 assay was used to evaluate the proliferation of tumor cells at 24 h, 48 h, and 72 h time points. No significant difference was observed among Hep3B-GSC, Hep3B-NC, and Hep3B cells. The data are presented as the mean ± SD of at least three independent experiments. Results were analyzed using Student's t test. (TIF) [file pone.0109695.s001.tif]
