# Supplementary material for: Goosecoid Promotes the Metastasis of Hepatocellular Carcinoma by Modulating the Epithelial-Mesenchymal Transition
Source: PLoS One. 2014 Oct 24;9(10):e109695. doi: 10.1371/journal.pone.0109695 (PMC4208742; doi:10.1371/journal.pone.0109695)
Supplement: Table S1 — Clinicopathologic factors and GSC expression in HCC. (DOC) [file pone.0109695.s002.doc]

**Table S1** Clinicopathologic factors and GSC expression in HCC

| **Clinicopathologic data** | **No. of patients** | | |  |
| --- | --- | --- | --- | --- |
|  | **GSC^Low^** | **GSC^Medium^** | **GSC^High^** |  |
|  | **(n=50)** | **(n=38)** | **(n=24)** | ***P*** |
| Age, y* |  |  |  |  |
| ≤60 | 40 | 34 | 19 | 0.446 |
| >60 | 10 | 4 | 5 |  |
| Gender* |  |  |  |  |
| Male | 43 | 33 | 23 | 0.443 |
| Female | 7 | 5 | 1 |  |
| HBsAg* |  |  |  |  |
| Negative | 8 | 8 | 4 | 0.813 |
| Positive | 42 | 30 | 20 |  |
| Cirrhosis |  |  |  |  |
| Absent | 37 | 30 | 20 | 0.643 |
| Present | 13 | 8 | 4 |  |
| AFP, µg/L |  |  |  |  |
| ≤20 | 17 | 13 | 7 | 0.932 |
| >20 | 33 | 25 | 17 |  |
| Tumor size |  |  |  |  |
| ≤5cm | 22 | 9 | 10 | 0.136 |
| >5cm | 28 | 29 | 14 |  |
| No. of tumor nodules* |  |  |  |  |
| Single | 37 | 31 | 21 | 0.386 |
| Multiple | 13 | 7 | 3 |  |
| Tumor capsule |  |  |  |  |
| Well encapsulated | 34 | 18 | 17 | 0.095 |
| Poorly encapsulated | 16 | 20 | 7 |  |
| Microvascular invasion |  |  |  |  |
| Negative | 38 | 25 | 12 | 0.086 |
| Positive | 12 | 13 | 12 |  |
| Edmondson grade |  |  |  |  |
| Grade(1/2) | 30 | 25 | 16 | 0.792 |
| Grade(3/4) | 20 | 13 | 8 |  |
| Portal lymphatic status* |  |  |  |  |
| No | 45 | 35 | 24 | 0.362 |
| Yes | 5 | 3 | 0 |  |
| Lung metastasis (Follow-up) |  |  |  |  |
| Yes | 17 | 23 | 16 | **0.009** |
| No | 33 | 15 | 8 |  |

HCC, hepatocellular carcinoma; No. number. *: Fisher’s exact test
